# Point of Care Technologies for HIV

**DOI:** 10.1155/2014/497046

**Published:** 2014-01-21

**Authors:** Mohan Kumar Haleyur Giri Setty, Indira K. Hewlett

**Affiliations:** Laboratory of Molecular Virology, Division of Emerging Transfusion Transmitted Diseases, Center for Biologics Evaluation and Research, Food and Drug Administration, Bethesda, MD 20892, USA

## Abstract

Effective prevention of HIV/AIDS requires early diagnosis, initiation of therapy, and regular plasma viral load monitoring of the infected individual. In addition, incidence estimation using accurate and sensitive assays is needed to facilitate HIV prevention efforts in the public health setting. Therefore, more affordable and accessible point-of-care (POC) technologies capable of providing early diagnosis, HIV viral load measurements, and CD4 counts in settings where HIV is most prevalent are needed to enable appropriate intervention strategies and ultimately stop transmission of the virus within these populations to achieve the future goal of an AIDS-free generation. This review discusses the available and emerging POC technologies for future application to these unmet public health needs.

## 1. Introduction

Acquired immunodeficiency syndrome (AIDS) is a disease of the human immune system caused by the HIV [1] which destroys CD4+ T lymphocytes of the immune system that prevent infections. AIDS is one of the most serious global health problems of unprecedented dimensions and is one of the greatest modern pandemics. At the end of 2010, an estimated 34 million people were living with HIV globally, including 3.4 million children under 15 years of age. There were 2.7 million new HIV infections in 2010, including 390 000 among children less than 15 years [2].

The annual number of people newly infected with the HIV has declined 20% from the global epidemic peak in 1998, mainly due to tremendous progress in diagnostics and treatment. Several diagnostic technologies have emerged with high specificity, sensitivity, and accuracy to detect HIV infection. HIV testing plays an important role in HIV prevention in that knowledge of HIV status has both individual and public health benefits. Early and accurate detection of HIV infection is important to public health because this stage is characterized by high infectiousness and transmissibility of the virus. The individual benefits of HIV testing are primarily associated with individuals accessing care and treatment. Individuals entering care and treatment have a substantial reduction in adverse health outcomes and increased life expectancy.

The past decades have witnessed enormous technological improvements towards the development of simple, cost-effective, and accurate rapid diagnostic tests for detection and identification of infectious pathogens. There is growing demand within the global health community to find ways to simplify and improve the efficiency of diagnostics for HIV/AIDS without diminishing the quality of patient care. At the same time, there is a need to significantly increase the level of access to robust, high-quality diagnostics in resource-limited settings in order to facilitate HIV prevention through early detection and treatment. Low-cost technologies to diagnose and monitor HIV infection in developing countries are a major subject of current research and health care in the developing world. With the great need to increase access to affordable HIV monitoring services in rural areas of developing countries, much work has been focused on the development of point-of-care (POC) technologies that are affordable, robust, easy to use, portable, and of sufficient quantitative accuracy to enable clinical decision making. For diagnosis of HIV infection, some low-cost tests, such as lateral flow tests and enzyme-linked immunosorbent assays, are already in place and well established. However, portable quantitative tests for rapid, POC, HIV monitoring have only recently been introduced to the market.

There is great need for initial diagnosis, staging, and ongoing monitoring of HIV. Among those that present the most persistent challenges to improved access and efficiency are CD4, viral load, and early infant diagnosis (EID). The great majority of testing options available today are laboratory-based platforms performed on sophisticated instrumentation requiring dedicated laboratory space and trained technicians. In many cases, laboratory-based testing is expensive; in almost all cases, it requires specimen transport networks to enable access for patients in periurban and rural settings. The success of high-quality antiretroviral therapy (ART) depends on simple, affordable, reliable, and quality-assured POC diagnostics for use in resource limited settings (RLS). POC diagnostics can make ART more scalable and will allow ART service delivery to be significantly decentralized to the community level. At the same time, simplifying diagnostic technologies that could potentially reduce the cost of diagnosing and monitoring patients living with HIV/AIDS without diminishing the quality of care is important for treatment success. The World Health Organization (WHO) has set criteria for at or near POC use—known as “ASSURED” criteria, meaning that they are (or will be) affordable, sensitive, specific, user-friendly, robust/rapid, equipment-free, and deliverable to those who need the test [3].

POC diagnostics are *in vitro* diagnostics (IVD) that do not involve the use of laboratory staff and facilities to provide the result. The analytical “targets” include proteins, nucleic acids, metabolites, drugs, dissolved ions and gases, human cells, and microbes. Test samples can be blood, saliva, urine, or other bodily fluids or (semi-) solids. Whether used “near patient” in a hospital, clinic, or doctor's office or administered at home to maintain health, manage disease, or monitor therapy or in the field to test the safety of water, food, or compliance with laws and regulations, these tests accept a sample with little or no preparation and provide a result, the “answer,” in seconds to hours [4].

The tests should require only elementary instruction to use and some detect multiple analytes or markers. Interpretation should be as simple as viewing a stripe or spot of color on a strip of paper or polymer; increasingly, however, readers ranging from hand-held devices to bench-top instruments read the analytical test, provide a comprehensible result, and if necessary, control and operate the sample-containing platform that executes the analytical process. These devices should be capable of using nanoliters to milliliters of complex biological media with femtomolar to millimolar concentrations of analytes [5]. The devices should be inexpensive disposable units or cartridges that include microfluidic features to provide or control sample preparation, flow rate, mixing with reagents, reaction time associated with binding events, filtration of nonanalytical components of the sample, separation of interfering agents and of multiple analytes, and an effective measurement capability [6].

“Why POC Diagnostics for HIV?” POC measurements provide results rapidly, where needed, and often with major time savings, samples do not travel to a laboratory to await the attention of a skilled technician; results do not wait to be transmitted and collected. Rather, the doctor, nurse, care-giver, patient, or consumer initiates the test and receives the results on the spot. Inevitably, this saves time, but speed must not be traded for accuracy or reliability.

## 2. HIV Diagnostics

Diagnostics for HIV/AIDS can generally be divided into three test categories: (i) tests to facilitate initial diagnosis, (ii) tests to stage the patient, and (iii) tests to monitor the patient, both before and after initiation of ART. There are generally accepted algorithms and tests used at each stage of the infection [7].

### 2.1. Low-Cost Diagnostic Tools for Monitoring HIV

Low-cost technologies to diagnose and monitor HIV infection in developing countries are a major subject of current research in health care settings in the developing world. With increasing need to provide access to affordable HIV monitoring services in rural areas of developing countries, much work has been focused on the development of point-of-care technologies that are affordable, robust, easy to use, and portable and of sufficient quantitative accuracy to enable clinical decision making. For diagnosis of HIV infection, some low-cost tests, such as lateral flow tests and enzyme-linked immunosorbent assays, are already in place and well established. However, portable, quantitative tests for rapid HIV monitoring at the point of care have only recently been introduced to the market [8]. The Ora Quick Rapid HIV-1/2 Antibody Test is a lateral flow rapid test for oral fluid specimens that perform as well as blood-based tests, even at low concentrations of HIV antigens in oral fluid [9]. The Aware HIV-1/2 U, with 97.2% sensitivity and 100% specificity, is a rapid alternative to urine tests that rely on ELISAs and Western blots.

Rapid HIV tests are expanding to include combination tests that detect both anti-HIV antibody and p24 antigen, as well as tests for early infant diagnosis and for HIV-1 and HIV-2 subtype differentiation [10]. Detection of both antibody and antigen during the acute phase of infection is particularly beneficial to prevention efforts because the rate of HIV transmission is several folds higher during this phase and can account for up to 50% of new HIV infections. The Determine HIV-1/2 Ag/Ab Combo is an immunochromatographic rapid test that detects antigen and antibody separately. In two independent studies, the Determine HIV-1/2 Ag/Ab Combo showed sensitivities of 50% and 86% for antigen detection [11, 12].

### 2.2. HIV Viral Load Monitoring

HIV viral load monitoring is crucial for therapy and staging of the disease. Reverse transcriptase (RT), an enzyme present during viral replication, correlates linearly with HIV load and CD4+ T-cell count [13]. Measuring this RT protein may be an alternative for resource limited settings, as its yielding results in a few hours and costs 80% less than nucleic acid tests [14].

Viral load estimates based on reverse transcriptase tests are not directly comparable to those obtained through PCR because they have been found to underquantify the HIV load [15, 16]. However, in places where PCR is unavailable, results of reverse transcriptase tests can be a cheaper viral load monitoring method. Further advantages of reverse transcriptase tests include their ability to detect multiple subtypes of HIV and their possible use in monitoring HIV load in pediatric patients [14]. Levels of the HIV core protein p24 also correlate with HIV load and CD4+ T-cell count. Assays for p24 have been shown to have lower sensitivity and there has been difficulty in correlating plasma p24 and viral RNA load in persons receiving ART [17]. However, improvements in sensitivity of p24 antigen tests may allow further reevaluation of these assays for HIV viral load monitoring.

#### 2.2.1. Current Viral Load Technologies

HIV viral load technologies can be categorized broadly as nucleic acid-based tests (NAT) and non-NAT-based technologies. NAT technologies detect and quantify viral RNA, whereas non-NAT technologies detect and quantify HIV viral enzymes and proteins that can be correlated with the amount of viral RNA. Most of the commercial assays use target amplification methods [3]. In general, the advantages of NAT-based approaches are their wide availability in quality-assured kits, and clinicians have familiarity with interpreting the results. The assays vary in terms of sample preparation and amplification/detection methodologies. The major NAT-based assays and platforms are discussed below [3].

There are four commercially available RT-PCR based viral load assays at present: (i) COBAS AMPLICOR HIV-1 MONITOR v1.5 (Roche Molecular Systems), (ii) COBAS AmpliPrep/COBAS TaqMan v2.0 (Roche Molecular Systems), (iii) Real-Time HIV-1 (Abbott), and (iv) VERSANT HIV-1 RNA 1.0 assay (kPCR) (Siemens). There are also a number of other in-house procedures and test systems that have good sensitivity and reproducibility and are used in various countries. The non-NAT-based technologies quantify proteins and enzymes specific to HIV. These include assays that measure the level of reverse transcriptase activity and assays that measure the concentration of circulating p24 protein [3].

#### 2.2.2. Reverse Transcriptase Technologies

Reverse transcriptase (RT) assays detect that viral enzyme, the RT activity can be quantified, and levels can be correlated with the amount of HIV. Therefore, an assay for RT can reflect the HIV viral load in the patient's blood. RT assays originally required radioisotopes, a scintillation counter, and an ultracentrifuge for performance, but they have been simplified and made less hazardous. Currently, there is one RT platform available for *in vitro* use—the ExaVir Load, manufactured by Cavidi AB [3, 18].

### 2.3. ExaVir Load (Cavidi AB)

Cavidi manufactures the ExaVir (Version 3), which is a quantitative HIV-RT test that is designed to measure viral-bound HIV RT activity in plasma in order to estimate the HIV viral load. The ExaVir Load assay is more manual than most of the other viral load assays described herein, but it is generally less expensive than other current molecular detection methods.

An advantage of the assay is that because the ExaVir Load determines viral load based on quantification of RT activity and does not target a specific nucleic acid sequence; it can measure any HIV type or subtype with high accuracy, including O and N groups. The measuring range of the assay is the equivalent of about 200 to 600,000 copies/mL (or 1 to 3,000 femtograms fg/mL). There is performance data available on the ExaVir Load showing good correlation with the COBAS AMPLICOR Monitor assay [3, 18].

#### 2.3.1. p24 Antigen Technologies

HIV-1 infection is characterized by an early spike in HIV-1 antigens in the blood. During this period of acute infection or antigenemia, the antigens in the blood are detectable, but in most individuals, the antigen levels subsequently become undetectable for a period of time. It is only later in HIV disease, with increasing failure of the patient's immune system and an increasing level of the virus, that the antigens may again become detectable in the blood. One of the viral components in blood during the period of antigenemia is the core protein, p24, the major internal structural protein of HIV-1. The p24 appears within 2 weeks after infection as a result of the initial increase in viral replication and is associated with the period of antigenemia during which the individual is highly infectious. Testing for p24 antigen can be of value in several circumstances: (i) detecting early HIV infection, (ii) diagnosing infection in infants (discussed later in this report), and (iii) monitoring ART. In the past, before the availability of NAT-based technologies, the p24 antigen assay was used for monitoring the development of AIDS and monitoring disease progression [19]. In particular, the NEN HIV-1 p24 ELISA assay from Perkin Elmer (an ultrasensitive, heat denatured p24 antigen quantification assay) has been used for this purpose. However, the p24 antigen tests available were not very sensitive and there are concerns about correlation of p24 with HIV RNA [20]. Moreover, with a linear range between 10,000 and 30,000 RNA copy equivalents/mL, the assay is of limited utility in detecting early treatment failure and it is not useful in patients with low viral replication [20]. Therefore, the use of p24 antigen testing will not be discussed further in this report in the context of monitoring patients on ART but will be revisited in the discussion of EID.

The latest laboratory immunoassays to come to the market for central laboratory use are antigen/antibody immunoassays, which can detect p24 antigen in addition to detecting anti-HIV, IgM and IgG. Combination antigen/antibody assays have been approved and used in many countries since the late 1990s [21–25]. These assays detect p24 antigen at the level of 11–18 pg/mL [26], which is equivalent to approximately 30,000–50,000 copies/mL of HIV RNA [27]. Recently, two such assays (Abbott ARCHITECT HIV Ag/Ab Combo and Bio-Rad GS HIV Combo Ag/Ab EIA) have been approved for use in the USA. Several studies have been conducted with these assays and the data indicate that they have similar performance characteristics as those marketed elsewhere [28–30] and detect p24 approximately 5–7 days after the appearance of nucleic acid [3]. Although these assays are central lab-based assays, they can be adopted to POC platforms for use in resource-limited settings as they are more sensitive and diagnose HIV infection earlier with a higher degree of accuracy.

#### 2.3.2. Viral Load Technologies for Future Use

The above NAT-based viral load systems described require testing to be performed in a laboratory setting at a central or national reference laboratory, by well-trained technicians. Although there are currently no POC viral load assays on the market, there are a number of platforms/assays in development. Described below are a few viral load assays that are currently being used and in the pipeline.

### 2.4. NAT System (Alere Inc.)

The Alere NAT system is a generic platform for the implementation of different NAT assays with an integrated platform for quantitative measurement of HIV-1 and HIV-2 viral load from approximately 25 *μ*L of whole blood. The system detects HIV-1 Groups M, N, and O and HIV-2. The device on which the assay is run (a prototype of which is pictured below) has a small footprint, is portable, battery-operated, and ruggedized to withstand harsh environments [3, 31].

### 2.5. Liat Analyser (IQuum, Inc.)

The Liat Analyser, manufactured by IQuum, Inc., is an automated sample-to-result NAT platform that performs sample nucleic acid extraction, purification, reverse transcription, polymerase chain reaction (PCR) amplification, and real-time detection to detect and/or quantify pathogens. Liat assays for HIV viral load testing and diagnostics have also been developed and independently evaluated by several laboratories. To aid the operator and provide reliable results, the Liat Analyser incorporates a variety of advanced features: barcode data entry avoids errors in sample or assay coding and on-screen prompts provide easy-to-follow directions to guide the operator through sample loading and tube insertion [3].

### 2.6. EOSCAPE-HIV HIV Rapid RNA Assay System (Wave 80 Biosciences)

Based on its EOSCAPE technology, Wave 80 Biosciences is developing a rapid HIV NAT-based POC viral load assay designed for use in resource-limited settings. The company describes the assay technology as incorporating non-PCR nucleic acid detection and quantitation, fingerstick whole blood processing within a single-use, enclosed cartridge. Using a fingerstick lancet, 100 *μ*L of whole blood is applied directly into the cartridge and no external sample preparation is required. The sample is automatically processed in 45 minutes; the operator then inserts the processing unit into the reader for a quick 2-minute scan. Equipped with a simple touchscreen interface, the reader is capable of transmitting test results through wired and wireless connectivity. The EOSCAPE-HIV system will be capable of providing either a qualitative or a quantitative HIV-1 RNA test result (detection threshold of 1,000 copies/mL) in less than an hour [3].

### 2.7. SAMBA (Diagnostics for the Real World)

The Simple Amplification Based Assay (SAMBA) is being developed by the Diagnostics Development Unit (DDU) at the University of Cambridge. Two NAT-based HIV assays are being developed: (i) a semiquantitative test for monitoring of ART and (ii) a qualitative test for the use in EID. The first SAMBA HIV assay to be launched will be the semiquantitative viral load assay. The SAMBA machine will integrate extraction, amplification, and detection into a bench-top analyzer with amplification and detection taking place in a closed cartridge [3].

The SAMBA HIV test uses 200 *μ*L of plasma or 100 *μ*L of whole blood. Amplification is based on both target and signal amplification. The visual detection of the RNA or DNA target can be read off of a test strip visually within 25 minutes. The test strip is based on a nitrocellulose membrane in a dipstick format. The SAMBA assay was able to detect all HIV-1 subtypes at 400 cp/mL. Currently, however, the SAMBA semiquantitative assay is calibrated to distinguish between patients with viral loads above or below 1000 cp/mL. The total amplification time of the SAMBA is one hour with throughput suitable for use at a small laboratory—for example, at district hospital level in sub-Saharan Africa. Diagnostics for the Real World, Ltd, the spinout company of DDU located in California, will be the manufacturer of the SAMBA system [3].

## 3. Additional Viral Load Technologies in the Pipeline

The Cepheid GeneXpert System, a fully automated and integrated system for PCR-based nucleic acid testing, is in use for detection of multiple pathogens and is being developed for HIV. The Northwestern Global Health Foundation (NWGHF) in collaboration with Quidel Corporation is developing a POC rapid RT-PCR testing platform that will be both easy to use and of low cost. Lumora has introduced the Bioluminescent Assay in Real-Time (BART), a platform for performing molecular diagnostics that allows real-time closed-tube quantitative detection of amplification by using a hardware system that can generate and store objective test results. Advanced Liquid Logic, Inc. (ALL) provides digital microfluidics technology solutions for liquid handling applications. The company has developed a compact bench-top immunoassay analyzer (pictured right) that is currently being evaluated. The company indicates that it is evaluating various assay formats as well as the portable analyzer. Cavidi is developing a new automated platform for near-patient viral load monitoring. The system will combine the strengths of RT technology with the advantages of an automated walk-away platform. This should provide fast and robust viral load monitoring for all HIV types and subtypes [3].

### 3.1. Early Infant Diagnosis

Diagnosing HIV infection early in infants can help in suitable therapeutic intervention which greatly reduces infant mortality due to HIV infection. Current p24 immunoassays have improved sensitivity compared with previous p24 tests because of the inclusion of a heat denaturation step for separating p24-antibody complexes that commonly interfere with assay sensitivity. Many studies support the use of the Perkin Ultra p24 immunoassay and dried blood spot analysis for early infant diagnosis (EID). However, p24 immunoassays are not widely used owing to a lack of validation data. Also, because p24 immunoassays require specialized equipment and skilled staff, p24 rapid tests are preferred for EID in resource-limited locations [32]. Development of p24 rapid tests has focused mainly on improving sensitivity [33]. In 2010, Parpia et al. tested a microfluidic p24 test on 25 *μ*L plasma samples from 389 South African infants [34]. Their design included a heat-shock component to break the antigen-antibody complexes. They achieved 95% sensitivity, 99% specificity, and a detection limit of 42 500 RNA copies/mL. The current prototype can be performed within 20 minutes.

### 3.2. Ultrasensitive p24 Antigen Assay (NWGHF)

NWGHF is developing an ultrasensitive p24 antigen rapid lateral flow assay for POC use. The technology, to be called Lynx, involves not only a lateral flow strip that detects HIV p24 antigen but preanalytical devices for separating plasma from heel-stick blood and disrupting immune complexes that would interfere with immunoassays. NWGHF has demonstrated proof of principle of the test. The assay procedure involves collecting 80 *μ*L of heel-stick blood from the infant using a capillary tube; plasma is separated and the sample is subjected to “heat shock” in small, battery-powered processor device; the rapid test strip is inserted into the device; and after approximately 30 minutes the result is read. The total assay duration is approximately 40 minutes. In early testing, the assay has shown about 95% sensitivity and 99% specificity.

### 3.3. PanNAT Diagnostic Platform (Micronics, Inc.)

Micronics, Inc., a subsidiary of Sony Corporation, has developed the PanNAT system, which is a small, portable microfluidic platform for POC use in *in vitro *molecular diagnosis of infectious diseases in resource-limited settings. It uses a fluorescent-based reader capable of processing individual, disposable, assay-specific cartridges, each of which is designed to perform a single- and/or multiplexed nucleic acid assay. The reader includes all necessary reagents on board. The system is lightweight, portable, battery- or centrally powered., WiFi-enabled, capable of storing up to 350 test results before prompting the user to download or delete results, and can provide results within 30 to 40 minutes.

*Portable Tests.* Portable tests for HIV monitoring comprise CD4+ T-cell count assays and HIV quantification assays. Currently, only a handful of truly portable CD4+ T-cell counters are available commercially: PointCare NOW, the CyFlow miniPOC (Partec), Pima Test (Alere), and Daktari CD4+ (Daktari Diagnostics). All are fully automated, can be powered by batteries or electricity, use ≤ 25 *μ*L of blood, and provide same-day results. Additional POC CD4+ T-cell tests in development include a multiplex infectious disease test (MBio Diagnostics) and a semiquantitative, electricity-free CD4+ T-cell blood test (Zyomyx) [35]. Both the PointCare NOW and CyFlow miniPOC measure absolute CD4+ T-cell counts and the percentage of T cells expressing CD4+ and do not require cold chain for reagent storage. The CyFlow miniPOC has a substantially high throughput, with the capability of processing 250 tests per day. No independent diagnostic data for either test were available at the time of writing. The Pima Test and Daktari CD4+ are cartridge-based tests in which a blood sample is inserted into a disposable cartridge containing the necessary reagents per test. The Pima Test, introduced in late 2010, has yielded results comparable to those of other ART monitoring devices [36–39]. While the majority of Pima Test measurements underestimate CD4+ T-cell counts, one study demonstrated 96.3% sensitivity for concentrations below a cut-off of 250 cells/*μ*L [38]. A recent study also found that Pima Test capillary blood samples yielded less precise results than venous blood samples. This highlights the need to address sources of error that can decrease a POC test's accuracy. No portable PCR tests for HIV quantification are currently on the market but several are in the pipeline.

Several other POC tests that may be portable are also under development. According to PATH, a low-cost version of a fully automated rapid PCR device is in development for resource-limited settings [35]. This test, known as the Liat HIV Quant Assay (IQuum Inc., Marlborough, USA), uses whole blood in a single container for PCR amplification and analysis in 88 minutes [40]. The SAMBA HIV-1 test is a dipstick-based nucleic acid assay for HIV detection and amplification. After collection of viral RNA, isothermal amplification of RNA is automated by a machine. The current prototype tests one sample in <2 hours but future prototypes are expected to test 5–10 samples simultaneously. In one study, SAMBA results agreed with RT- PCR results for 69 clinical samples with different viral subtypes or viral loads (7.8 to 9.5 × 10^6^ copies/mL). Additional advantages of this test include (i) its lower cost because, unlike PCR, it requires no thermo cyclers; (ii) that it has multiplexing capability, which permits detection of HIV subtypes; and (iii) that it allows same-day diagnosis. An additional SAMBA test for qualitative early infant diagnosis is also under development. Other companies developing POC viral load tests include Alere and Micronics. Several other POC PCR platforms in development might also be easily adapted for HIV-associated health care [35].

## 4. HIV Screening in Resource-Limited Settings

The most ready-to-use types of rapid tests are lateral flow tests, which do not require complex reagents. In general, rapid tests are less expensive (less than 1 United States dollar [US$] per capita), portable and easy to use, require few or no reagents or equipment, and provide results within 30 minutes. Each test rapidly detects antibodies against HIV from a small volume of plasma, serum, whole blood, saliva, or urine, with high specificity and sensitivity. The use of saliva and urine specimens for rapid testing offers a discrete alternative to blood-based tests in settings where stigma, lack of education, cultural practices, and privacy concerns undermine HIV prevention [8]. In addition, the noninvasiveness of specimen collection eliminates the anxiety associated with blood collection and leads to higher rates of voluntary HIV testing [41].

### 4.1. CD4 Counts

The measurement of the absolute CD4 T-cell count is critical in the initial evaluation and staging of HIV-infected persons. HIV infects primarily immune CD4+ T lymphocytes (CD4+ cells) [42]. Antiretroviral therapy, ART, especially when CD4+ cells are not yet depleted, can reduce viremia and slow its progression to AIDS [43]. Furthermore, if CD4 counts are established, antiretroviral prophylaxis can reduce the risk of transmission in pregnant women to prevent mother-to-child transmission of HIV during pregnancy, childbirth, and breastfeeding [44]. Therefore it is critical to perform CD4+ cell counts upon diagnosis of HIV and before initiating ART. The WHO currently recommends ART initiation if absolute CD4 counts are below 350/cells mm3 [44]. The performance of the diagnostic platform with respect to accuracy and precision is the most important for using such technologies. This is particularly challenging for CD4 testing platforms as “no gold standard technology or internationally recognized reference preparation exists for CD4” [45]. Misclassification may delay the initiation of ART or prophylactic treatment in some patients or treat large numbers of patients who have CD4 counts above the ART initiation threshold.

### 4.2. Existing CD4 Technologies/Platforms

There are currently a handful of platforms that account for CD4 testing in resource-limited settings. These are lab-based single platform systems from BD Biosciences, a division of Becton Dickinson (BD), Beckman Coulter (Coulter), Millipore (formerly Guava and now a division of Merck), Partec, and Apogee. In the developing world, BD and Coulter are the most commonly used platforms for CD4 testing [45].

### 4.3. Point of Care CD4 Testing Platforms

The above-mentioned high-, medium-, and low-throughput platforms are systems primarily designed for use in laboratory settings. A number of them, including the FACSCalibur, Epics and FACSCount, are used in developed as well as resource-limited settings. The CD4 assays developed include selective cell staining, followed by capture or count by digital photography, measuring CD4 molecules instead of cells, or measuring proxy molecules of CD4. Some of the POC technologies are already in use. They include PointCare NOW, the Pima CD4 Analyser, and the CyFlow CD4 miniPOC. The remaining technologies including Daktari, Burnet, Zyomyx, MBio, and others are not yet commercially available [3].

### 4.4. Other Possible CD4 POC Tests

In addition to the POC CD4 tests/devices discussed above, there are a few other research and development groups working on platforms/devices that could potentially be used for CD4 counting.

### 4.5. Automated POC CD4 Test Devices

There are several commercial POC CD4 counting devices that are now available and some are under development. Most of them use finger-stick blood samples, which are rapid, are robust, have flexible power options, and utilize stable, dried reagents enclosed in a single test cassette or device. The devices are designed for minimal operator use with varying throughput result outputs. The PointCare NOW and the CyFlow miniPOC are modified flow cytometers. The PointCare NOW is the only POC CD4 device commercially available. The system is significantly heavier than the other technologies making it less portable and venipuncture is required for larger specimen volumes, which may limit its use in low resource settings where phlebotomists are scarce. However, additional equipment such as pipettors is not needed for operation and therefore supply of other critical reagents is not a factor.

A CD4 POC test marketed by Alere (Waltham, MA), the Pima CD4 test, is the first commercially available automated CD4 counting method that does not use traditional flow cytometric approaches. The Pima utilizes dual-fluorescence image analysis to count CD3+ and CD4+ using labeled anti-hCD3 and anti-hCD4. The Pima cartridge contains all reagents and specimen during processing. The Pima reader supplies the pumping functions and images the results to determine colocalization of CD3+ and CD4+ cells. The result expressed as the absolute number of CD4+ T lymphocytes per *μ*L and the CD3+/CD4+ ratio is displayed by the instrument along with quality control results. Cartridges using beads that represent “Normal” and “Low” counts are intended for daily quality control testing of the PIMA instrument. Recent evaluations in Zimbabwe and Mozambique have shown good performance in comparison to flow cytometry and user assessment of the test protocols that suggest that the Pima has the potential for use at the POC [36, 46].

Daktari Diagnostics, Inc. (Boston, MA) is developing a CD4 test that uses a novel microfluidic affinity-chromatography/shear-gradient technique to differentially capture CD4+ cells from whole blood and a unique nonoptical detection to count them. The test consists of a reader and a disposable cassette which contains all of the reagents, stabilized in blister packs. Here, flowing CD4+ cells adhere to an antibody-coated chamber while larger-sized monocytes are subject to large shear forces and cannot bind. The captured CD4 cells are then lysed and the release of cellular ions is measured by impedance spectroscopy [47]. The decrease in impedance in the chamber due to CD4+ cell binding has been shown to correlate with cell count over three orders of magnitude, including 350 CD4+/mm3, the threshold for ART [48].

mBio Diagnostics, Inc. (Boulder, CO) is developing a CD4+ cell-counting system, SnapCount. It is a static two-color fluorescence imaging cytometry system composed of single-use disposable cartridges and a simple reader with on-cartridge immune staining of whole blood samples. The instrument addresses the high cost of conventional optical systems by using LightDeck technology, a fluorescence assay illumination approach that is a variation on planar waveguide technology which uses low-cost lasers, optics, and imaging sensors that are now ubiquitous in cell phones and consumer electronics. As the instrument is only utilized in the reading step, multiple cartridges can be processed in parallel, providing a throughput of eight to ten samples per hour. This relatively high sample throughput might allow remote health care settings to meet their greater demand with fewer instruments [49].

### 4.6. Nonautomated CD4 Test Devices

In 2005, the CD4 initiative held by Imperial College, London, (funded by the Bill & Melinda Gates Foundation) partnered with industrial and academic teams to develop power-free POC CD4 tests [50] and three devices are now being further developed from this project. The Burnet Institute CD4 test device is a semiquantitative immune chromatographic strip (ICS). Some ICS assays have been shown to be effective tools for POC diagnostics in RLS; they are small, of low cost, have acceptable performance and reagent stability, and have a test format that is familiar to the intended user group. The Burnet CD4 test has been incorporated into a lateral flow strip (similar to an HIV rapid diagnostic test) with traditional rapid test format, including monocyte removal pad and immunogold. In this device the transmembrane domain of cellular CD4 is recognized by biotin-labeled anti-hCD4 which is detected by a colloidal gold labeled anti-biotin. A capture stripe of anti-CD4 is adjacent to a reference stripe of a biotinylated surrogate protein. The CD4 count is determined by the user to be either greater or lower than the reference stripe.

Beckman Coulter has been developing a similar semiquantitative ICS format to enumerate CD4 at the POC. Zyomyx, Inc. (Hayward, CA) has developed a novel bead sedimentation system to count CD4+ cells [19]. The reagents include high-density anti-hCD4 particles as well as anti-hCD14 magnetic beads. The device uses sedimentation of high-density, bead-associated CD4+ cells as a measurement principle. The height of the sedimented and visible bead/cell column in a capillary is directly proportional to the number of CD4+ cells when viewed against a precalibrated scale, much like reading a thermometer [19].

There is no technology currently in widespread use for counting CD4 at the POC. Most of the technologies described above have incorporated the basic tenets for POC in terms of instrument size, specimen volume, reagent stability, independent power supply, and simple user interface. However, other factors such as throughput, associated consumables and reagents, user training, robustness of design, and the method of analysis vary considerably.

Three assays using either scaled flow cytometric methods (PointCareNOW and the CyFlow miniPOC) or novel technologies (the Alere Pima) are also in use. Flow-based systems have the advantage of established technology and have been thoroughly evaluated in larger scale formats. Thus, evaluation of the new instruments will focus on improving and demonstrating the robustness and the ease of use in RLS. The need for appropriate quality controls (QC) and POC testing is an area where QC is essential to ensure that specimen preparation, equipment, and reagents are functional, for user compliance with the protocol. The Pima incorporates QC indicators for specimen collection and reagent and equipment function. The devices being developed by Daktari and mBio offer cassette-based instrumented readers for increased accuracy with a simple user interface and single or no moving parts. This simplified instrumentation is anticipated to cost less than other CD4 instruments in addition to having potentially lower failure rates and reliance on maintenance [49].

### 4.7. Viral Load Testing Technologies Overview

Viral load testing is the method favored for monitoring HIV patients once they have been initiated onto ART. High levels of HIV circulating in the bloodstream indicate that the virus is actively replicating, and these levels can be used, with the aid of molecular methods, to provide important information regarding the risk of disease progression and to predict the outcome of infection [19]. Viral load testing is complicated by HIV diversity and certain practical challenges, including laboratory infrastructure and transport of samples.

HIV is classified into two HIV-1 and HIV-2 major types. Of the two types of HIV, HIV-1 is predominant and has been most responsible for the HIV pandemic that exists today [19]. HIV-1 is divided into four groups, designated by M, N, O, and P, the main group of which is group M. Within the HIV-1 group M, nine clades are recognized, designated by the letters A–D, F–H, J, and K. Recombinants between different HIV-1 group M subtypes are designated as either circulating recombinant forms (CRFs) if fully sequenced and found in three or more epidemiologically unlinked individuals or as unique recombinant forms (URFs) if not meeting these criteria [51]. Up to 55 CRFs have been described so far [51, 52]. Some CRFs have recombined further with other subtypes or CRFs giving rise to the so-called second-generation recombinants (SGRs).

The enormous diversity found in HIV as discussed earlier and extraordinary ability of this virus to evolve continuously pose great diagnostic challenges. The high level of genetic heterogeneity of HIV-1 and the emergence of recombinant strains of the virus complicate viral load assay development. In an ideal world, viral load assays would detect and quantify all known HIV-1 subtypes as well as inter-subtype recombinants and emerging variations thereon. But currently, that is not the case, although the assays are able to recognize most HIV-1 subtypes. Therefore, it is important to consider the prevalence of HIV-1 and HIV-2 groups and subtypes in a particular geographical region when choosing a viral load assay.

### 4.8. PCR-Based Diagnostics

In response to the HIV/AIDS crisis, access to antiretroviral therapy (ART) has increased dramatically over the past decade in low- and middle-income countries [53]. However, successful management of HIV requires that patients receiving ART be monitored routinely to assess treatment efficacy and detect treatment failure due to drug resistance and other causes. Unfortunately, current laboratory-based methods to monitor ART are unaffordable, unavailable, or inappropriate for low-resource settings [54].

Rapid antibody tests are widely available in developing nations, but they cannot be used to monitor HIV progression or treatment efficacy. The standard of care to monitor ART is quantitative viral load testing based on plasma HIV RNA concentration [55]. Although CD4 count has also been used to monitor ART, recent studies suggest that it may not detect early treatment failure adequately [56]. The gold standard method for viral load testing, RT-qPCR, is unsuitable for settings where trained technicians, expensive reagents, electrically powered equipment, and dedicated laboratory space are often unavailable. Therefore, a viral load test that is appropriate for such settings is needed.

The NATs have the potential to offer many advantages at the POC, such as low limits of detection and quantification of the level of infection. PCR is an enzyme-driven process for amplifying short regions of DNA *in vitro*. It can create millions of DNA copies by cycling between different temperatures to allow repeating steps (denaturation, annealing, and elongation) of DNA replication to take place. Despite the simplicity and amplification power of PCR chemistry, limitations in its supporting hardware still hinder PCR from reaching its full potential. In particular, improvements in thermal cycling speed, instrument size, and reaction volume are still much needed. The bulky instrumentation and large reaction volume required in conventional bench-top thermal cyclers lead to large thermal mass which reduces the temperature transition speed and reaction efficiency.

Rapid tests are widely used to screen for HIV infection at POC and this has significantly expanded diagnostic capabilities of testing sites in developed countries, as well as resource-limited settings. Despite advances made by the widespread availability of rapid tests, all antibody-based detection of HIV exhibits some limitations. HIV-specific antibody typically begins to appear around three weeks after infection, allowing for detection by most antibody-based assays within 3–6 weeks [57]. This window period prior to or during early sero conversion may lead to false-negative test results in recently infected individuals. Additionally, accurate diagnosis of infants born to HIV-infected mothers can be challenging if based solely on antibody positivity, since vertically transferred maternal antibodies may persist for 12–18 months after birth [58]. For confirmatory diagnosis of early HIV infection or infant diagnosis, nucleic acid amplification tests (NAAT) are preferred, as HIV-1 RNA can be detected as early as 10–12 days after infection and HIV-1 DNA and/or RNA are definitive indicators of active infection [59]. In their current form, however, NAATs are not feasible for POC testing, because they are time consuming, expensive, and technically complicated.

## 5. Current Isothermal Amplification Technologies

### 5.1. Isothermal Amplification

Isothermal amplification techniques operate at a single temperature, eliminating the need for a thermocycler, enabling them to be conducted on simple and portable heating systems. Several isothermal amplification techniques have been developed in recent years, such as helicase-dependent amplification, rolling circle amplification, and nicking enzyme amplification reaction [60–62]. Loop-mediated isothermal amplification (LAMP) [10] has been optimized for the detection of DNA and/or RNA (RT-LAMP) from a wide range of bacterial and viral pathogens including HIV [63–65].

LAMP or RT-LAMP exhibits several characteristics that are ideal for integration into a rapid nucleic acid-based diagnostic test. The amplification reaction requires six primers specific for eight separate regions within the target sequence, contributing to the high specificity of the amplification method. Amplified material can typically be detected within 15–60 minutes when incubated at a constant reaction temperature of 60–65°C [66]. LAMP has also proven to be less sensitive to biological inhibitors than PCR [67], which enables direct amplification from clinical specimens, thereby eliminating the need for an additional nucleic acid extraction step. Direct amplification from plasma, whole blood, and oral fluid has previously been demonstrated for HIV-1 [63, 64, 68]. Lastly, immediate visual detection of amplified products is facilitated by the large amount of DNA that is generated by each reaction. Several groups have incorporated fluorescent detection methods into the LAMP assay for real-time or immediate naked-eye detection [64, 66, 69].

### 5.2. Nucleic Acid Sequence-Based Amplification (NASBA)

The NASBA techniques like transcription mediated amplification (TMA) and self-sustained sequence replication (3SR) mimic *in vivo* retroviral replication mechanisms to produce RNA amplicons from an RNA template. Modified cDNA is formed from an RNA template, which is then rapidly amplified into RNA amplicons. The complete process is a single step and proceeds in a single volume with an ssRNA product suited for direct use with hybridization probes making NASBA very appealing for point-of-care Technologies (POCT) [70].

The target RNA being labile calls for faster and careful sampling procedures as RNA may degrade. Lower incubation temperature is desirable as it will reduce the power consumption and thermal control complexity but may result in a low stringency reaction environment and allow nonspecific amplification, making robust primer design and assay evaluation crucial [71]. Furthermore, an initial 95°C strand separation step is required if dsDNA is to be targeted whilst RNA amplification requires a 65°C step to remove secondary structures. These temperature steps will be an engineering consideration in POCT devices where precise thermal control and associated increased power consumption will be disadvantageous.

### 5.3. Helicase-Dependent Amplification (HDA)

The high temperature requirement to separate dsDNA can be overcome by using stand separating enzyme helicase in PCR amplification of DNA. The typical existing HDA protocol needs 60–120 min for low copy number targets. This shortcoming can be overcome by including the use of restriction endonucleases targeting regions upstream of the target sequence to enhance helicase activity in the target region, addition of crowding agents, and increasing enzyme concentrations [20]. The HDA is appealing for POCT as it requires single set of primers and two enzymes (three for reverse transcription-HDA), and is compatible for existing fluorescent detection chemistries [20].

#### 5.3.1. Recombinase Polymerase Amplification (RPA)

RPA is a single tube, single temperature (37–42°C) amplification method. The key to the amplification process is the formation of a recombinase filament, a complex combining a target-specific primer, and a recombinase enzyme. When the target-specific sequence is encountered by the recombinase filament, it performs strand exchange, inserting the primer onto the target. The displaced d-loop formed is stabilized by ssDNA binding proteins (gp32) to prevent reannealing. Spontaneous disassembly of the recombinase filament upon strand exchange leaves the primer/target hybrid open to extension by strand-displacing polymerase activity. Repetition of the cycle leads to geometric amplification. Favorable thermal requirements, procedural simplicity, and very rapid amplification (20–40 min) make this recently developed process [72] a leading technology for integration into POCT devices. The added “off-temperature” ability of RPA to proceed at a variety of temperatures is of great appeal for field applications where precise temperature control is often technically challenging and will allow for instrument-free amplification. The biochemistry of RPA is incompatible with existing intercalating dyes, molecular beacons, and TaqMan technology. Alternative fluorescent probe detection strategies like Twist-Ampexo and TwistAmp have been developed to allow single tube fluorescent detection using sequence-specific probes. As an emerging technology, there is comparatively little in the published literature regarding RPA technology, RPA primer/probe design, and its integration with POCT devices.

#### 5.3.2. Loop-Mediated Isothermal Amplification (LAMP)

Among the isothermal nucleic acid methods currently available, loop-mediated isothermal amplification [73] (LAMP) is the most widely researched and has been well characterized offering significant support during the development process. LAMP is a rapid amplification method employing a strand displacing Bst DNA polymerase and 4–6 primers, two of which are “fold back” primers [74] which form stem-loops motifs with self-priming capability. This results in an amplification scheme where the priming sequence is copied with each round of replication and remains tethered to the previous amplicon resulting in a concatenated product of alternating sense/antisense repeats of varied length. Subsequent studies have found that the use of additional “loop primers,” which bind to the loop structures, can greatly reduce the reaction times resulting in a total of 6 primers [75].

The 60–65°C reaction temperature combined with a minimum of 4 primers makes LAMP a highly specific reaction allowing an “amplification is detection” scheme. This specificity has allowed the insoluble pyrophosphate reaction by-product [76] to be employed in a turbidimetric detection strategy for both qualitative visual indication [77] or real-time quantitative turbidimetry [78], which offers a very simple, robust detection strategy for POCT integration. Whilst a 95°C initial strand separation step is not essential [79], it has been shown to increase analytical sensitivity [80]. As with some other isothermal methods (SMAP, NEAR, and SDA), LAMP is highly dependent on the careful design of multiple complex primers and this can be overcome by the use of appropriate software [74].

#### 5.3.3. Rolling Circle Amplification Technology (RCA)

RCA is a powerful technique which exploits the strand displacement and highly processive polymerase activity of the Phi29 bacteriophage DNA polymerase (Q29DNAP) acting on circular DNA targets [81, 82]. The basic RCA reaction (linear RCA or single primer RCA) is initiated by a primer annealing to a circular ssDNA. The Q29DNAP can elongate a new strand of the circular template eventually completing a loop and reaching the point of initiation. Strand displacement activity allows the newly forming strand to continuously displace the previously generated strand as polymerization advances. Generation of a continuous catenated ssDNA of up to 0.5 mega bases [83] has been reported and continues until an external factor, such as nucleotide depletion, halts the reaction. This continuous catenated product attached to the template allows in situ or localized amplification, which can be used to concentrate labels within a small detection area and enumerating single DNA molecules [61, 84, 85].

#### 5.3.4. Single Primer Isothermal Amplification (SPIA)

SPIA is a linear amplification technology for DNA based on repeated replication of target sequences enabled by the use of chimeric RNA/DNA primers, which bind target regions and initiate polymerization [86]. The RNA/DNA primer is engineered in such a way that RNase H degradation of the RNA portion of the chimeric primer will reexpose the binding site to allow a subsequent primer to anneal. Strand displacement activity of the polymerase removes the previously generated strand. This repeated cycle continuously generates new amplicons until reagents or primers are depleted. A similar method, Ribo-SPIA, developed to amplify total mRNA, replicates only the original transcripts and not copies, resulting in a high-fidelity product. SPIA will become more widespread as genomic analysis enters the point-of-care domain.

#### 5.3.5. Smart Amplification Process Version 2 (SMAP2/SmartAmp2)

This nascent amplification technology (not to be confused with the signal amplification method SMART, signal-mediated amplification of RNA technology) employs similar enzymes and self-priming loop motifs to LAMP. In contrast to the symmetrical primers of LAMP, SMAP2 primers are designed asymmetrically with different tail motifs in the two target flanking primers. This serves to reduce the formation of background products from misamplification. The amplification process occurs in two steps: an initial “key intermediate” step forming a target sequence flanked 3′ and 5′ with fold-back domains to provide self-priming ability and a second amplification step where the key intermediates undergo repeated self-priming and rapid target amplification resulting in concatenated, primer inclusive amplification products.

In addition to this amplification format, SMAP2 employs background suppression technology to increase specificity and permit an “amplification is detection” assay. Ultrahigh specificity is achieved by employing Thermus aquaticus MutS to identify mismatched primer/target hybrids. MutS scans dsDNA and will irreversibly bind to any mismatch duplex with single nucleotide sensitivity [87]. Bound MutS prevents polymerization thus checking amplification of nonspecific sequences resulting in complete inhibition of nonspecific amplification. The incorporation of MutS and asymmetric primer design permits single nucleotide discrimination, making SMAP2 particularly useful for SNP identification. Whilst SMAP2 has been shown to proceed slower than LAMP, the SMAP2 technique's very high specificity, high sensitivity (3 copies), and powerful amplification (100-fold larger than PCR) combined with the developers (DNAFORM and RIKEN, Yokohama, Japan) reporting of specific detection from crude cell lysate make this a promising development and potentially powerful tool for POCT devices [88, 89].

#### 5.3.6. Strand Displacement Amplification (SDA)

This method relies on bifunctional primers incorporating both target recognition and endonucleases regions [90, 91]. Following strand separation, these bifunctional primers extend incorporating the restriction target into the amplicon. Bumper primers, which bind and extend upstream, release this amplicon. Successive rounds of primer binding generate dsDNA incorporating restriction sites, which can then be acted upon by the restriction endonucleases to nick a single strand of the newly formed duplex. This nicking allows the polymerase to displace the existing strand and incorporate a new amplicon. This nick and run scheme is repeated to effect exponential amplification. Single strand nicking is effected by the incorporation of a modified adenine nucleotide, dATP aS (59-O-l-thiotriphosphate), which is resistant to the endonuclease activity. Thus only the newly incorporated primer will be nicked leaving the amplified strand to repeatedly act as a template for primer binding. The complex asynchronous reactions occur concurrently and user interventions are limited to an initial heat denaturation with primers followed by addition of polymerase and restriction enzymes at a 37 uC190 incubation, a protocol which is by no means complex and appears amenable to POCT use [91].

#### 5.3.7. Nicking and Extension Amplification Reaction (NEAR)

NEAR is a recent development of the earlier described EXPAR reaction [62]. Capitalizing on nicking enzymes to expose binding sites for primers, the EXPAR displays excellent reaction kinetics and 10^6^- to 10^9^-fold amplification in a few minutes. However, EXPAR is limited to amplification of sequences adjacent to native nicking enzyme recognition sites within the target genome [92]. NEAR is a refinement of EXPAR to allow amplification of any target by inserting nicking-enzyme recognition sites adjacent to target regions. The two-stage NEAR reaction proceeds in a similar manner to the SDA reaction exploiting nicking-enzymes to generate a site from which polymerase elongation can initiate. In contrast to SDA, the nicking enzyme employed in NEAR will only nick a single side of a duplex, removing the need, as seen in SDA, for strand modification of the duplex to prevent double stranded cleavage.

#### 5.3.8. Isothermal and Chimeric Primer-Initiated Amplification of Nucleic Acids (ICAN)

ICAN is a simple scheme for DNA amplification at 55°C using relatively few reagents: a pair of 5′-DNA-RNA-3′ chimeric primers, thermostable RNase H, and a strand-displacing DNA polymerase [93]. Following initial heat denaturation of the target dsDNA, the chimeric primer binds to the template and is elongated by BcaBEST DNA polymerase. The newly formed strand is nicked by thermostable RNase H action, not at the 3′ border of the chimeric primer as initially thought but at the penultimate 3′ RNA residue, allowing the strand displacing DNA polymerase to release a newly synthesized strand with a single 5′ RNA residue, leaving the template with a truncated primer, which is still sufficient to prime elongation. The cycle repeats until the chimeric primer is sufficiently shortened, allowing a new, free chimeric primer to anneal preferentially, recommencing the cycle [94].

In addition to this multipriming model, a template switching mode of amplification has been identified [95]. Template-switching amplification occurs when both forward and reverse primer bind to the same dsDNA target and proceed to elongate toward one another, eventually switching the template from using the original template to using the newly synthesized strand elongating from the opposite primer as the template. This displaces both parent strands, forming a dsDNA of two daughter strands consisting of the target flanked by the primer regions with incorporated chimeric primer on one strand. This dsDNA of daughter strands is acted upon by RNase H, which introduces a nick in the RNA region and polymerase elongation can commence. If both forward and reverse elongation reactions occur simultaneously, the template switching cycle will recommence with the parent strands being displaced and the chimeric primer-bound strands becoming the dsDNA product. If the nicking and elongation occur asynchronously, a single-stranded product is formed, still having an incorporated chimeric primer. This single strand with primer can enter the multipriming amplification cycle; thus there is no dead-end product and amplification will be sustained until the reagents become exhausted.

#### 5.3.9. Conclusion

Many of the isothermal amplification methods described above display speed, amplification power, and analytical and diagnostic specificity and sensitivity equal to, and often in excess of, existing molecular techniques based on real-time PCR/qPCR. Given this excellent performance and their suitability for miniaturization, it is highly likely that isothermal amplification strategies will become commonplace in the next generation of point-of-care diagnostic devices. Although tremendous progress has been made since the discovery of PCR, still there is no robust nucleic acid-based point of care PCR.

## 6. Microfluidic Diagnostics

This section categorizes various fluidics technologies such as pressure-driven flows, capillary flows, electromagnetically driven flows, centrifugal fluidics, acoustically driven flows, and droplet fluidics. Then three broad categories of POC microfluidic testing devices are considered: lateral flow devices, desktop and handheld POC diagnostic platforms, and emergent molecular diagnostic POC systems. Such evolving trends as miniaturization, multiplexing, networking, new more sensitive detection schemes, and the importance of sample processing are discussed. It is concluded that POC microfluidic diagnostics have a potential to improve patient treatment outcome and bring substantial savings in overall healthcare costs.

Microfluidic diagnostics had an explosive growth in the last 20 years spurred by the convergence of clinical diagnostic techniques (such as blood gas analysis, immunoassays, and molecular biology testing) and mature micro fabrication technology that allowed production of sub-millimeter-size fluidic channels and reservoirs in a variety of material systems (e.g., silicon, polydimethylsiloxane (PDMS), and poly (methyl methacrylate) (PMMA)) [96].

### 6.1. Microfluidic Technologies

Microfluidic diagnostics use technologies to accomplish a predetermined set of operations (i.e., to bring the sample and reagents together, to add buffer, to implement wash, and to facilitate the readout) required by the specific biochemistry of the tests and detection techniques. They can be classified according to fluid propulsion such as pressure-driven flow and electromagnetically driven flow. They can also be classified according to the type of flow like continuous flow or the so-called segmented flow (where fluid is advanced in discrete packets or droplets). The segmented flows (also called droplet microfluidics), an extremely important emerging technology, can be achieved on a variety of platforms, for example, centrifugal platforms, pressure driven platforms, and electromagnetically driven flows.

Strip-based tests that use capillary forces are among the most ubiquitous and commercially successful POC tests. There are many evolving LF testing technologies like nucleic acid hybridization-based LF devices [97] or a combination of antibody-antigen recognition with nucleic acid hybridization in nucleic acid lateral flow immunoassays [98] and utilization of advanced labels such as resonance-enhanced absorption [99], chemiluminescence [100], upconverting phosphors [101], silver-enhanced gold nanoparticle labels [102]. Other trends involve developing a larger number of immunoassays [103], advancing quantification of the detection [104], and quality control in manufacturing to increase reproducibility of tests.

### 6.2. A Lateral Flow Assay for Quantitative Detection of Amplified HIV-1 RNA

Rebecca R. Richards-Kortum's group from Rice University, Houston, TX, have described a lateral flow assay that employs gold nanoparticle probes and gold enhancement solution to detect amplified HIV RNA quantitatively. They showed, coupled with nucleic acid sequence-based amplification (NASBA), that this assay can detect concentrations of HIV RNA that match the clinically relevant range of viral loads found in HIV patients. Their lateral flow assay achieved a resolution of 0.5 log_10_ copies/mL over a linear range that extends 2.5 orders of magnitude ranging from 10.5 to 13 log_10_ RNA copies. When coupled with NASBA, the LFA can detect 50 copies of HIV gag RNA. We speculate that this LOD corresponds to a plasma viral load of roughly 1000 copies/mL, assuming that a plasma sample volume of 100 mL will be used and that half of viral RNA from the sample is added to the NASBA reaction. The performance of the LFA when detecting NASBA products suggests that the LFA may be sufficient to detect significant changes in viral load, suppression of viral replication, and therapeutic failure. The LFA only requires a heat block, scanner or camera, and pipette. The lateral flow assay uses a sample volume of 20 mL, requires only three steps over the course of 20 minutes, costs $0.80 per strip using commercial reagents, and performs consistently after short-term storage. By modifying the target capture, positive control, and probe sequences, the LFA may be adapted to detect other RNA targets. The LFA is capable of detecting short, amplified sequences or long, genomic sequences. The LFA is suitable for low-resource settings and has the potential to be performed at the POC [105].

### 6.3. Miniaturization of NAT Instrumentation

Miniaturization of NAT instrumentation has been the focus in recent times; Genie II (OptiGene Horsham, UK, and Twista (TwistDx, Cambridge, UK) demonstrates the possibility for more portable NAT equipment. [106]. Separate amplification and detection of the PCR product allow for greater sensitivity and elimination of nonspecific signals. With regard to POCT applications, the requirement of multiple procedural steps for amplification and separate detection is cumbersome. However, development of simplified assays is limited by the need to maintain analytical sensitivity and specificity in a protocol consisting of fewer steps.

### 6.4. Combined Amplification and Detection

The advent of fluorescent DNA probes and intercalating dyes [107, 108] has allowed the real-time quantification of amplification products in both PCR and isothermal amplification reactions. The limiting factor of such systems is the need to maintain analytical sensitivity to the target whilst developing reaction conditions with sufficiently high analytical specificity to eliminate nonspecific products accumulating and producing a false-positive result. The use of sequence-specific fluorescent probes such as TaqMan and molecular beacons offers the benefits of combined amplification and detection in reactions lacking the specificity for nonspecific detection methods. Probe-based real-time detections also allow for multiplexing using different probe fluorophores. The simplicity of combined amplification and detection in a single step is desirable and will hasten the time to result.

### 6.5. Amplification-Free Direct Nucleic Acid Detection

Such techniques use highly sensitive detection strategies to identify target sequences in a sample without the need for nucleic acid amplification. This offers the potential for simplified protocols, reduced reagent consumption, and simplified operating platforms. The success of such systems is entirely dependent on the development of robust biosensors with analytical sensitivity sufficient to detect the very low abundance NA in complex clinical samples whilst maintaining appropriate clinical specificity for diagnostic use. Non-POCT systems incorporating direct NA detection are commercially available. For example, NanoString (Seattle, USA) offers a bench-top instrument using molecular barcodes for gene analysis. Portable systems for direct detection, such as those being developed by Genefluidics (Irwindale, USA) using electrochemical detection and ExoCyte (Reading, UK) employing silicon nanowire, carbon nanotube, and quantum dot technologies, have yet to be cleared for diagnostic use. Direct detection technologies are expected to enter the global testing market within the next 10 years and have the potential to displace numerous methods currently in use [106].

## 7. Paper-Based Sensors

Paper is an extremely versatile material possessing desirable properties such as mechanical flexibility and strength. The main constituent of paper is cellulose fiber, and this can be highly attractive for certain applications as it allows liquid to penetrate within its hydrophilic fiber matrix without the need of an active pump or external source [109]. Moreover, cellulose fibers can be functionalized, thus changing properties such as hydrophilicity, if desired, as well as its permeability and reactivity. It can be produced sustainably and inexpensively, is ubiquitous globally, and provides an excellent substrate for chemical fictionalization, making it well suited to use in low-cost, point-of-care (POC) diagnostic devices. In recent times, paper has been employed in the construction of more complicated analytical devices known as lab-on-a-chip (LOC). Conventional LOC devices often made of glass or PDMS, while considerably more simplified than their full-scale laboratory counterparts, still maintain a cost which is prohibitive to their deployment in economically developing regions due to the specialist equipment and clean rooms required for their manufacture [110]. In 1956, the first paper device for the semiquantitative detection of glucose in urine was demonstrated [111], that further developed into immune chromatographic paper test strips (also known as lateral flow or dipstick tests), with the pregnancy test kit being a well-known example [103]. Paper has been suitably modified to detect analytes by different methods like colorimetric, electrochemical, electrical conductivity, chemiluminescence, and electro-chemiluminescence [112].

Paper-based sensors are a new alternative technology for fabricating simple, low-cost, portable, and disposable analytical devices for many application areas including clinical diagnosis, food quality control, and environmental monitoring. The unique properties of paper which allow passive liquid transport and compatibility with chemicals/biochemicals are the main advantages of using paper as a sensing platform. Depending on the main goal to be achieved in paper-based sensors, the fabrication methods and the analysis techniques can be tuned to fulfill the needs of the end user. Current paper-based sensors are focused on microfluidic delivery of solution to the detection site whereas more advanced designs involve complex 3D geometries based on the same microfluidic principles. Although paper-based sensors are very promising, they still suffer from certain limitations such as accuracy and sensitivity. However, it is anticipated that in the future, with advances in fabrication and analytical techniques, there will be more new and innovative developments in paper-based sensors. These sensors could better meet the current objectives of a viable low-cost and portable device in addition to offering high sensitivity and selectivity and multiple analyte discrimination.

### 7.1. CMOS Cell Sensors for Point-of-Care Diagnostics

Complementary metal-oxide-semiconductors- (CMOS) based products can enable clinical tests in a fast, simple, safe, and reliable manner, with improved sensitivities. Extensive use of CMOS-based sensors in DNA diagnostics [113, 114] and commercialization of CMOS-based DNA sequencing systems (Ion Torrent Systems) [115] is already promising for the involvement of CMOS-based cell detection systems in medical applications.

### 7.2. Challenges in Paper Platforms

Although there is enormous potential in paper as a platform for LOC devices, there is a lot of scope to improve in terms of sensitivity. This is typically due to how the sample is introduced onto the paper, which can be at a considerable distance of 10 to 15 millimeters from the detection zone. As the analyte is delivered across the paper, the local analyte concentration may decrease as a result of solution spreading and may also evaporate if the distance and time of travel are far from the point of introduction. The other problems are encountered in multiplex analysis as there is the potential for cross-talk as some of the signal reporters may diffuse to neighboring channels [112]. There are limited chemistries available for the conjugation of biomolecules to cellulose for use in biomedical applications.

### 7.3. HIV Subtypes Isolation on Chip

Utkan Demirci group have demonstrated a microfluidic chip device that can effectively capture various subtypes of HIV particles through anti-gp120 antibodies, which were immobilized on the microchannel surface. They showed that Protein G-based surface chemistry has a better control over the antibody orientation on the surface compared to antibody immobilization methods including passive adsorption, covalent binding, and NeutrAvidin-based surface chemistry. This platform technology can be potentially used to measure HIV-1 viral load in resource-constrained settings. Protein G-based surface chemistry when used together with NeutrAvidin-based surface chemistry enables the separation of capture and detection chemistries that can potentially reduce the nonspecific binding and enhance detection outcomes Their immunosensing device enables the development of POC on-chip technologies to monitor viral load and guide antiretroviral treatment (ART) in resource-constrained settings [116].

## 8. Fraunhofer ivD-Platform

A consortium of seven Fraunhofer Institutes have developed a lab-on-chip system called “Fraunhofer ivD-platform” which can be potentially used as POC diagnostics. The platform features a high degree of modularity and integration. Modularity allows the adaption of common and established assay types of various formats. Integration lets the system move from the laboratory to the point-of-care settings with multiplexing capability. By making use of the microarray format, the lab-on-chip system also addresses new trends in biomedicine. The low-cost device has reagents reservoirs, microfluidic actuators, and various sensors integrated within the cartridge. In combination with fully automated instrumentation (read-out and processing unit), a diagnostic assay can be performed in about 15 minutes. This is possible with user-friendly interfacing read-out unit, data acquisition, and data analysis units together.

The assays for nucleic acids (detection of different pathogens) and protein markers (such as CRP and PSA) have been done using an electrochemical sensor based on redox cycling or an optical sensor based on total internal reflectance fluorescence (TIRF). Furthermore, integration of sample preparation and polymerase chain reaction (PCR) on-chip has been done. The instrument is capable of providing heating-and-cooling cycles necessary for DNA amplification [117].

## 9. Detection

Electrochemical and optical technologies are the clear leaders in detection technologies in the market as they do not use complicated instrumentation for detection. Therefore point-of-care detection should be able to detect signal without using any complicated instrumentation or if the results can be visualized.

### 9.1. Optical Detection

Optical detection is the simplest and most popular method used in immunoassay applications. Most immunosensors are based on optical detection and commonly use a label (e.g., a fluorescent label, enzyme, or metallic particle) that may facilitate optical signal enhancement and increase detection sensitivity. Optical immunosensors combined with microfluidic chips have recently been proposed as an attractive immunosensing platform, and many reviews have explored their potential applications in clinical diagnostics, particularly because there is a growing need to simultaneously screen multiple proteins in a single sample. The optical detection method, which can be easily implemented in microfluidic systems, is a prime candidate for this multiplexed analysis. Optical detection methods can be divided into five main categories on the basis of detection signal: fluorescence, luminescence, absorbance (colorimetry), surface plasmon resonance (SPR), and surface-enhanced Raman scattering (SERS); each technique has inherent advantages and disadvantages. In this section, we focus on the recent multiplexing applications in microfluidic immunosensors using optical detection techniques.

There are several optical detection methods used for POC applications such as fluorescence with variants such as Forster resonance energy transfer (FRET) and upconverting phosphor technology, luminescence, absorbance (colorimetry), surface-plasmon resonance (SPR), and various categories of light scattering: Rayleigh (particles much smaller than wavelength), Mie (particles comparable to wavelength, shape dependent), geometric (particles larger than wavelength), resonant (wavelength overlaps an electronic transition of the particle), and Raman (vibrational quanta added to or subtracted from the excitation wavelength) [118]. The most commonly used technique is absorbance as it is commonly used in LFAs based on gold or polymer (nano-) particles. However fluorescence is used for the broadest range of different types of POC assays [119] for reasons of sensitivity and, more recently, the ready availability of a range of different colors of efficient fluorophores, including quantum dots, quantum-dot barcodes, and fluorescent nanoparticles, providing improved limits of detection. Fluorescence in some cases can detect single particle LODs enabling multitarget multiplexing [120].

The supercritical angle fluorescence (SAF) which is being used recently detects fluorescence emitted in close proximity to a fluorophore-supporting optically transparent chip surface. This method provides substantial enhancement of fluorescence collection efficiency while rejecting background from unbound fluors or impurities, as it confines the fluorescence detection volume to material within about one wavelength of the chip surface [121].

Generally the analyte is labeled by attaching a chromophore, fluorophore, or particle (dye containing, semiconductor/quantum dot, noble metal, or scattering) to an antibody or nucleic acid strand that confers specific recognition for optical detection. Nanoparticles including quantum dots are finding increasing application. Nanoparticle labels conjugated with biomolecules have been used in a variety of different assay application. Nanoparticles offer adjustable and expandable reactive surface area compared to the more traditional solid phase forms utilized in bioaffinity assays due to the high surface-to-volume ratio. Signal enhancement by conjugating nanoparticles with fluorescent, luminescent, and other measurable properties has enhanced detection limit by several folds. The potential to multiplex along with the ability to increase sensitivity and specificity without using enzymes has increased the use of nanoparticles in immunoassays for early detection. It has been shown that using time resolved fluorescence of lanthanides like europium nanoparticles with long stokes shift can reduce background and increase detection limit to as low as 0.5 pg/mL of HIV p24 [122]. The determination of cancer biomarkers in serum and saliva using quantum dot bioconjugate labels are used. Quantum dots were employed on chip for CD4+ T-cell counting in a POC application. Aptamers were tethered to gold nanoparticles as part of an LFA-like dry-reagent assay strip to detect thrombin [118]. Thermal-lens microscopy (TLM), an alternative to fluorescence detection, also uses dye labeling for detection. TLM detection integrated to a miniaturized ELISA device along with optical, electronic, and fluidic components could detect an LOD of 2 ng/mL for total IgE measurement [123].

### 9.2. Electrochemical Detection

#### 9.2.1. Electrochemical Immunosensors

Electrochemical immunoassays are the most commonly used analytical techniques for the quantitative detection of biomolecules, followed by optical methods. Electrochemical immunosensors are not only sufficient to meet the demands for sensitive, rapid, and selective determination of analytes but can also be incorporated into robust, portable, and miniaturized devices. Specifically, the integration of electrochemical detection with microfluidic chips offers an attractive immunoassay platform with significant advantages derived from the combination of electrochemical analysis and a microfluidic system [124]. In broad terms, electrochemical immunosensors function by detecting an electrical signal that arises from specific immunoreactions that occur at the surface of an electrode. According to the type of electrical detection signal, electrochemical techniques are classified into three basic categories: voltammetry (current), potentiometry (potential shift), and impedimetry (resistance) [125].

Some analytes are electroactive and can be measured directly without labeling; electrochemical detection often utilizes tagging for analyte specificity with either an electroactive species or an enzyme that converts an electrochemically silent species into electroactive one; this approach also provides signal amplification of multiple orders of magnitude, with detection limits below 1 pM readily accessible [118, 126]. It has been shown that ultrasensitive capacitive immunosensor is capable of detecting subattogram per milliliter concentrations of p24 antigen [127].

#### 9.2.2. Piezoelectric Immunosensors

Piezoelectric devices convert a physical or mechanical change into electrical energy and vice versa. The commonest piezoelectric sensor is the quartz crystal microbalance (QCM), which exploits the change in the resonance of quartz crystals upon changes in their mass, allowing binding of antigen to antibody (when one of these is immobilised on the crystal surface) to be measured electrically [128].

#### 9.2.3. Microcantilevers (MC)

Sensors based on MCs are an attractive method for sensitive and label-free detection. On the basis of advances in micro fabrication techniques, massively parallel cantilevers can be facilitated in microfluidic devices to independently monitor multiple targets [129]. Two distinct modes of MC immunosensors that are utilized for the signal transduction of immune recognition binding events on the MC surface are the deflection (cantilever bending) and resonance (resonant frequency shift) modes.

Microcantilever-based devices were developed utilizing rotating resonance microcantilevers which measured the frequency-shift of the microcantilever motion with respect to the specific adsorbed mass, to give sensors capable of measuring *α*-fetoprotein to less than 2 ng mL for the label-free, POC for early detection of hepatocellular carcinoma [130]. Also a microring resonator immunosensor has been recently described which can detect multiple analytes (PSA, *α*-fetoprotein CEA, tumor necrosis factor-*α* and interleukin-8) concurrently, without loss of sensitivity or measurement precision when compared to single-parameter analysis [131].

### 9.3. Magnetic Detection

Magnetic particles are widely used for POC diagnostics because they can be used to preconcentrate and they can also be used as a labeling technology for detection without the requirements of fluors for optical transparency. Magnetic particle detection technology has evolved rapidly, the most promising and sensitive methods now using the giant magnetoresistance (GMR) effect with detectors based on the so-called spin valve (SV) or magnetic tunnel junction (MTJ) methods [132].

#### 9.3.1. Label-Free Methods

The label-free methods for POC detection include SPR, amperometric immunosensor, and electrochemical sensors based on mechanical transduction and direct electrochemical and optical transduction for analytes possessing suitable characteristics.

Silicon nanowire (SiNW) biosensors have been extensively studied in the last decade for the detection of biological molecules as highly sensitive, label-free, and electrical tools. SiNW biosensors hold great promise to realize POC devices for disease diagnostics with potential for miniaturization and integration [133].

Optical surface plasmon resonance (SPR) biosensors represent the most advanced and developed optical label-free biosensor technology. Optical SPR biosensors are a powerful detection and analysis tool that have vast applications in infectious disease diagnostics [134].

Mechanical transducers for POC applications oscillate or resonate, including micro- and nanocantilevers [135], as well as various acoustic wave devices such as the quartz-crystal microbalance (QCM) and a range of devices in the surface acoustic wave family [136]. Operating characteristics such as frequency and signal attenuation for piezoelectric devices or resistance and amplitude for piezoresistive (silicon) devices are affected by the mass and mechanical properties of molecules and materials linked to their oscillating surfaces, like SPR; they require only an immobilized selective recognition layer. Nonetheless, “mass tags,” dense particles (typically Au) that bind selectively to the target, can significantly enhance sensitivity. The “bond rupture sensors,” use acoustic energy to rupture bonds between immobilized capture antibodies and target microbes. Because the energy and frequency of added energy can be adjusted, nonspecifically and specifically captured particles can be desorbed differentially according to the bond strengths and masses, providing a unique discrimination mechanism [137].

#### 9.3.2. Integrated Rapid-Diagnostic-Test Reader Platform on a Cell Phone

Aydogan Ozcan group have demonstrated a cell phone-based rapid-diagnostic-test (RDT) reader platform that can work with various lateral flow immuno-chromatographic assays and similar tests to sense the presence of a target analyte in a sample. This compact and cost-effective digital RDT reader, weighing only 65 g, mechanically attaches to the existing camera unit of a cell phone, where various types of RDTs can be inserted to be imaged in reflection or transmission modes under light-emitting diode- (LED-) based illumination. Captured raw images of these tests are then digitally processed (within less than 0.2 seconds per image) through a smart application running on the cell phone for validation of the RDT, as well as for automated reading of its diagnostic result. The same smart application then transmits the resulting data, together with the RDT images and other related information (e.g., demographic data), to a central server, which presents the diagnostic results on a world map through geotagging. This dynamic spatiotemporal map of various RDT results can then be viewed and shared using internet browsers or through the same cell phone application. They have tested this platform on malaria, tuberculosis (TB), and HIV RDTs by installing it on both Android-based smart phones and an iPhone [138].

#### 9.3.3. Plasmonic ELISA

Molly M. Steven's group have demonstrated an ultrasensitive ELISA, which uses a signal-generation mechanism for biosensing that enables the detection of a few molecules of analyte with the naked eye. The enzyme label of an ELISA controls the growth of gold nanoparticles and generates colored solutions with distinct tonality when the analyte is present. HIV-1 capsid antigen p24 was detected in whole serum at the ultralow concentration of 1×10^−18 ^g/mL of p24 and was also detected with the naked eye in the sera of HIV-infected patients, showing viral loads undetectable by a gold standard nucleic acid-based test [139].

## 10. Opportunities and Challenges for Cost-Efficient Implementation of New Point-of-Care Diagnostics for HIV

High-quality diagnostics are essential to fight against HIV and to reduce its transmission. There are clear diagnostic needs where conventional laboratory support is insufficient and not cost effective. HIV rapid point-of-care (POC) testing for initial HIV diagnosis has been successful, but several needs remain. Despite its clear advantages, POC testing has important limitations, and laboratory-based testing will continue to be an important component of future diagnostic networks [140].

Many middle- and low-income countries implementation of rapid, cost-effective, POC HIV diagnostic screening have developed algorithms for diagnosing HIV infection using strategic orthogonal testing assays without the need for additional confirmatory testing [141]. Use of saliva has broadened the testing but saliva samples have been associated with higher false-positive rates in some testing sites [142]. Despite the advances and successes of HIV serological rapid assays, unmet gaps for qualitative POC applications remain. First, we need to identify acute HIV infection because recently infected individuals are seronegative but have extremely high viral loads, making them far more infectious compared with chronically infected individuals [143]. Early detection can result in early treatment initiation, which limits disease progression and reduces transmission [144].

Second, we need to diagnose infants born to HIV-infected mothers, particularly in sub-Saharan Africa. Due to the placental transfer of antibodies during pregnancy, infants may test positive on serological assays yet may not be infected with HIV [145]. Currently, infant diagnosis is primarily performed by collecting dried blood spots and transporting the samples to central testing laboratories for DNA testing [146]. However, many infants go undiagnosed because of long turnaround times or insufficient infrastructure (e.g., unreliable sample transportation) [145].

Third, we need appropriate tests for individuals vaccinated with experimental HIV antigens in clinical trials [147, 148]. Vaccine volunteers mount specific immune responses to the vaccine constructs, which react with many serological diagnostic tests, making future HIV diagnosis difficult, potentially unblinding trial staff, and negatively impacting society [148]. Similar assays may also be beneficial in resource-limited settings to monitor antiretroviral therapy (ART) effectiveness and the potential need to switch therapy [149]. In each of these cases, new rapid molecular POC HIV screening tests will fill an important diagnostic gap.

## 11. Future Trends

Strip-based tests with the use of low-cost polymer substrates, like paper and thread microfluidics, and low-cost readers like smart phones are also expected to facilitate home testing. Digital/droplet microfluidic devices are being actively researched and only recently have begun to enter the field of POC diagnostics will become popular. The gap between “traditional” (e.g., porous nitrocellulose) lateral flow assay devices and microfluidic platforms is narrowing with the development of these technologies. While PCR remains a gold standard for high sensitivity and specificity in most cases, there are other emerging related isothermal PCR techniques to consider. There are a range of related PCR techniques such as RCA, LAMP, NASBA, MDA, TMA, SDA, and LCA (rolling-circle amplification, loop-mediated isothermal amplification, nucleic acid sequence-based amplification, multiple-displacement amplification, transcription-mediated amplification, strand-displacement amplification, and ligase chain reaction, resp.), as well as cleavase Invader which have the potential to be used in POC settings. Importantly, many of these methods are isothermal and operate at lower temperatures than PCR, making their integration with microfluidic technologies more straightforward. For detection, new optical methods exploit phenomena including upconversion, high-brightness nanoparticles, total-internal-reflection fluorescence (TIRF), SAF, FRET, and a range of plasmon-based effects. In many cases, nanoparticles enhance optical detection. The above technologies enhance signal, reduce background, or both; combined with low-cost, compact high-intensity solid-state light sources, this drives POC LODs even lower. Detecting multiple analytes in a single POC test is an important trend; many of the most promising new POC opportunities are in multianalyte tests or panels.

## 12. Conclusion

This review has outlined some of the key developments in the field of point-of-care technologies currently in use for HIV/AIDS as well as technologies that hold promise and currently under development for future use in improving early infant diagnosis and viral load monitoring, particularly in resource limited settings. The timely development of these testing approaches will be critical to ushering in a new era of AIDS prevention with its goal of an “AIDS-free” generation. The feasibility of achieving this goal will be greatly enhanced by implementing targeted improvements in testing and developing new therapies for HIV/AIDS. In recent years there has been significant involvement of both private and public sectors in the development of diagnostic technologies to meet the need for HIV testing in resource-limited settings. Future strategies may utilize the most appropriate technologies to help achieve prevention goals for HIV/AIDS through sustainable impact in the public health setting.
